# Case report: Report of a case of female adnexal malignant tumor of Wolffian origin

**DOI:** 10.3389/fonc.2024.1458817

**Published:** 2024-09-16

**Authors:** Shujun Ji, Xiao Yu, Yufang Xia, Yifan Yin, Tingting Ge, Lihua Cheng, Cui Tian, Yanhui Lou

**Affiliations:** Department of Obstetrics & Gynecology, The Affiliated Hospital of Qingdao University, Qingdao, China

**Keywords:** female adnexal tumors, Wolffian tumors, tubal malignancies, targeted therapy, immunotherapy

## Abstract

A 33-year-old young woman with a rare female appendage tumor of suspected Wolffian origin was initially diagnosed with a benign lesion after the resection of a tubal lesion due to the benign cytomorphology of the tumor tissue. However, 1 year after surgery, she was diagnosed with stage IV fallopian tube cancer due to a recurrence, which presented with substantial ascites and invasion of multiple organs, including the bilateral ovaries, intestines, pelvic peritoneum, greater omentum, and appendix. After tumor cytoreduction, the patient responded well to treatment, which included a regimen of platinum-based drugs combined with docetaxel, aromatase inhibitors such as letrozole, antihormonal therapy, and targeted therapy with bevacizumab.

## Case presentation

On Lunar 4, 2021, a 33-year-old patient, presented to our hospital with a pelvic mass discovered half a month earlier. Tumor markers tests revealed normal levels for CA125 (42.9 U/mL), on April 29, 2021, a laparoscopic debulking of the left tubal mesosalpinx tumor was performed. During the operation, a cystic solid mass measuring approximately 8 × 6 cm was identified in the left tubal lining and completely excised. The postoperative pathology findings revealed (left fallopian tube mesentery) the following: solid sheets or microcystic structures, glandular arrangement, mild cellular heterogeneity, occasional nuclear schizophrenia, focal tumor growth around blood vessels, consistent cell morphology and size, and visible nuclear grooves. The provisional diagnosis suggested gonadal-mesenchymal tumors, with a consideration of adult-type granulosa cell tumors, measuring 3 × 2 × 0.8 cm. Immunohistochemistry results showed the following findings: ER (estrogen receptor, ++, 60%), PR (progesterone receptor, ++, 60%), CKpan (+), Ki-67 (+, 20%), Calretinin (+), CD10 (partially +), and CD10 (partial +), Vimentin (+), β-Catenin (membrane +), Inhibinα (+). Due to the mild cell morphology and insignificant tumor heterogeneity observed under the microscope, the diagnosis was difficult. Following pathology consultations with the University of Pittsburgh Medical Center (UPMC) and the People’s Hospital of Peking University, a female adnexal tumor (Wolffian tumor) of mesonephric origin was considered. This diagnosis was based on the patient’s young age and nulliparous status, and the absence of heterogeneity in the morphology of the tumor cells under the microscope. The tumor was completely excised during the operation, and the patient was advised to undergo regular postoperative reviews and follow-up appointments. The patient had no abnormality in the whole abdomen enhanced computed tomography (CT) in March after surgery and positron emission tomography (PET)-CT in June after surgery.

**Figure 1:**
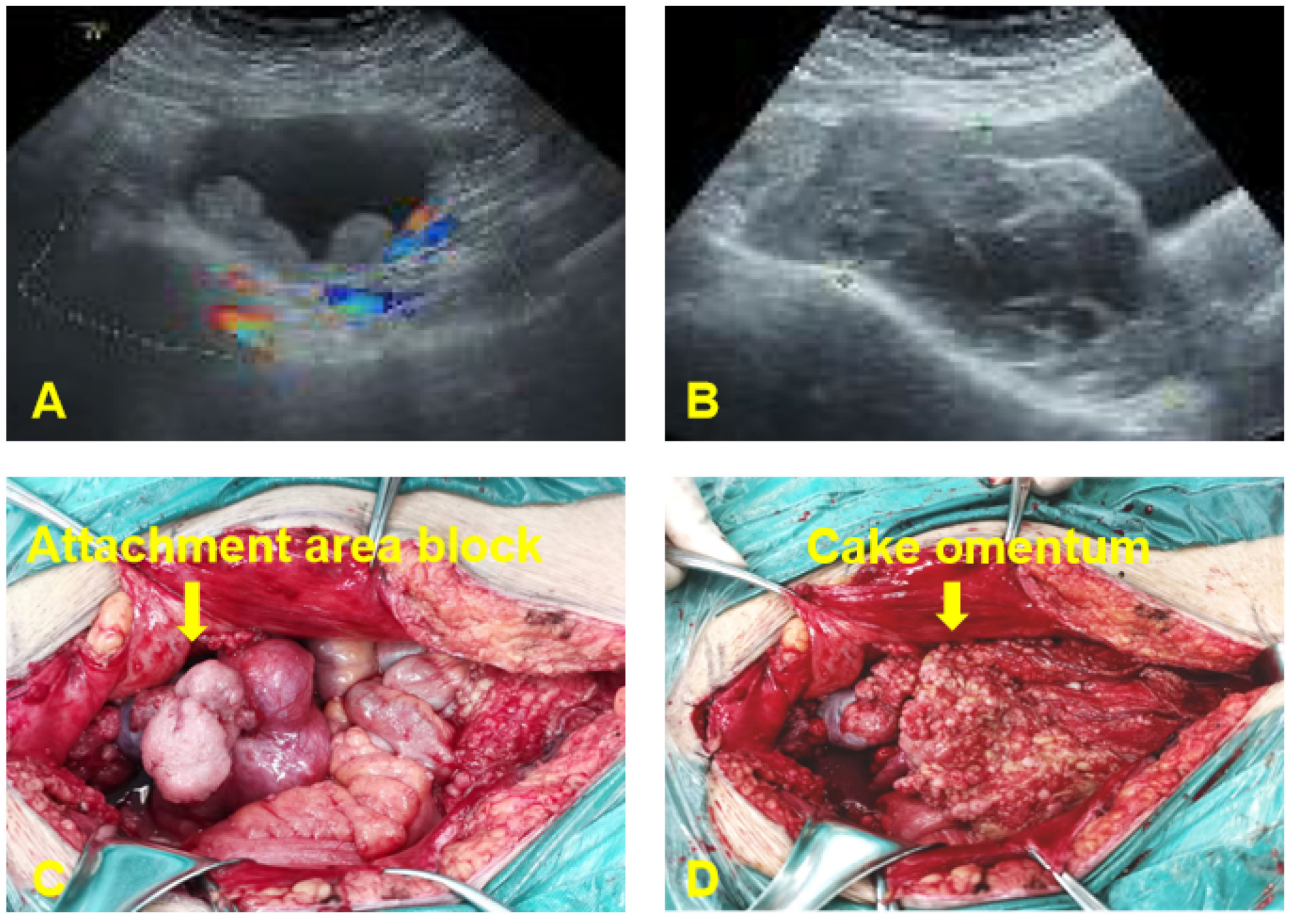
Preoperative ultrasound examination and intraoperative investigation of the patient. **(A)** Lunar 4, 2021: A 7.0*7.6 cm cystic mass was found in the upper left part of the uterus, with a thick cyst wall and multiple slightly hyperechoic protrusions. **(B)** May 10, 2022: A 12.8x10.1x5.8 cm solid cystic mass with a detached and multilocular cystic portion was present in the right adnexal region. The mass crosses the anterior aspect of the uterus and involves the left ovary. **(C)** Exophytic granular masses in the adnexal region bilaterally. **(D)** Granular metastatic nodules in the greater omentum.

**Figure 2:**
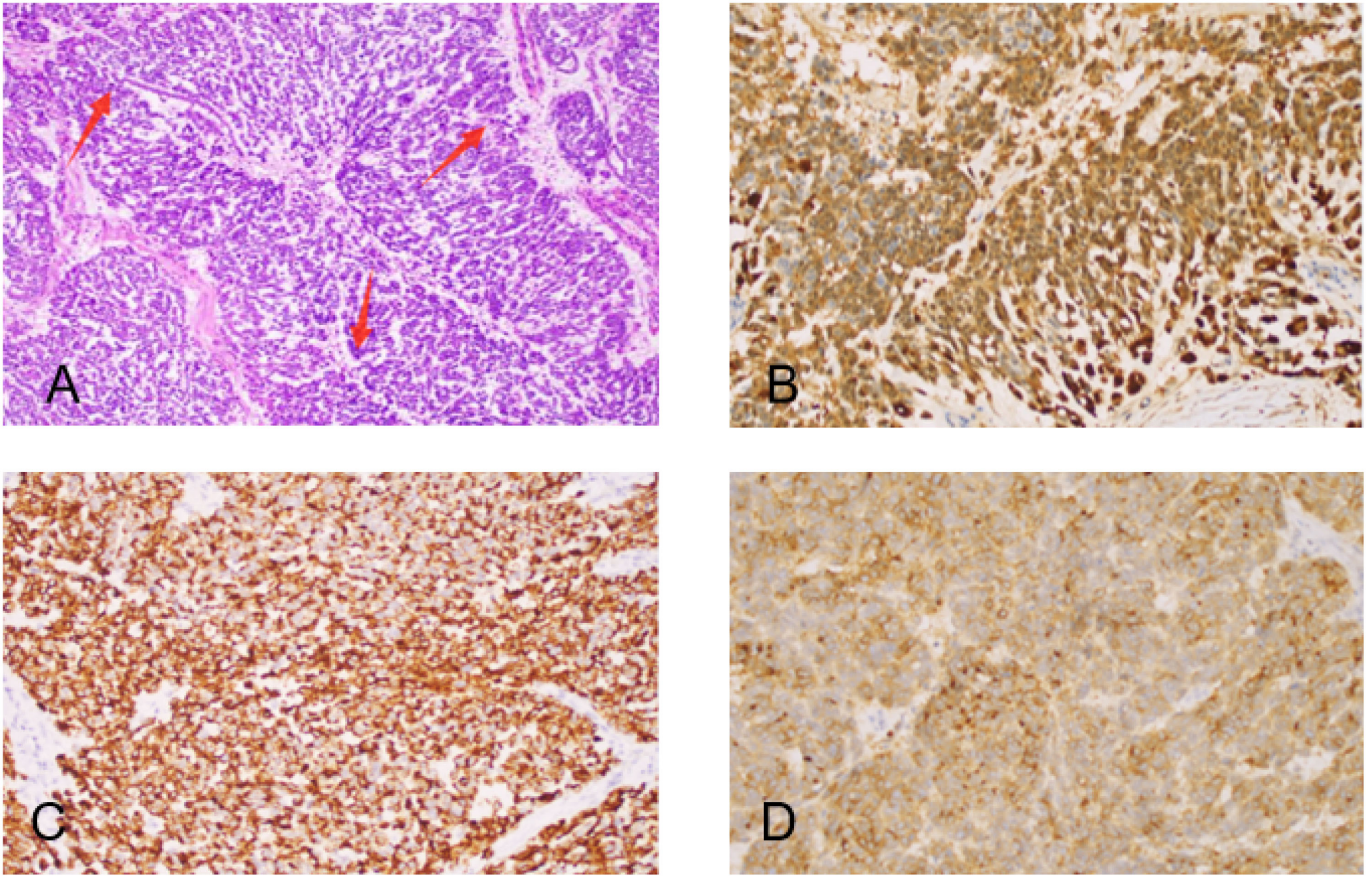
Hematoxylin-eosin staining and Immunohistochemical staining (both 400+). **(A)** High magnification micrograph showing medium-sized oval tumor cells with eosinophilic cytoplasm, atypical nuclei, prominent nucleoli, pronounced nuclear grooves, and increased mitotic activity (red arrowheads). **(B)** Calretinin positivity. **(C)** focal CK positivity. **(D)** CD10+ positivity.

One year after surgery, due to sudden abdominal distension, the patient underwent a whole abdomen CT, which revealed multiple malignant tumors in the pelvis, indicating extensive metastasis to retroperitoneal lymph nodes, peritoneum, and greater omentum. A chest CT revealed enlarged lymph nodes in the right cardiophrenic angle area, with potential metastasis; the tumor was considered to be recurrent with malignant changes. Tumor marker levels were elevated CA125(453.9 U/mL). On May 10, 2022, an open laparotomy was performed. During the operation, approximately 4000 mL of reddish ascites was seen in the pelvis. The size of the uterine corpus was normal with scattered granular nodules on the surface. An exophytic granular mass approximately 12 cm in diameter was seen in the right adnexal area, and an 8 cm diameter mass in the left adnexal area, with histological structures that were difficult to recognize and no typical tubal-ovarian tissues detected. Furthermore, a 6 cm diameter granular mass was detected in the uterine rectal sulcus. The peritoneum of the uterine rectal recess and the bladder and uterus exhibited nodular thickening. The greater omentum was markedly thickened with granular nodules scattered across its surface. The omentum of the splenic region was markedly thickened, and 2 cm metastatic nodules were seen at the lower level of the spleen. The appendix was markedly thickened, with granular nodules visible on its surface. Furthermore, granular nodules were observed throughout the pelvic and abdominal peritoneum, at the diaphragmatic apex, in the hepatic and renal crypts, along the hepatic ligament, the perihepatic peritoneum, the perisplenic peritoneum, the small bowel, the colon, and mesenteric membrane, ranging 0.2-3 cm in diameter. Granular nodules, most of which were loosely attached to the metastatic site, and enlarged lymph nodes of approximately 5 × 4 cm in size were found on the left side of the abdominal aorta and below the renal vessels, which were fixed, and no obvious enlargement of the pelvic lymph nodes was detected as shown in **Figure 1**. During intraoperative cryopathology, neoplastic lesions were identified both in the left and right adnexa. These lesions exhibited morphology similar to the previously diagnosed left tubal mesenchymal tumors but with increased heterogeneity and prominent nuclear fission. The opinion was malignant, and female adnexal tumors of mesonephric (Wolffian duct) origin or gonadal-mesenchymal tumors (with malignant biological behavior) were considered. Since the patient was infertile, after explaining her condition to her family, she underwent tumor cytoreductive surgery (R1). This included bilateral adnexectomy, total extrafascial hysterectomy, lymph node dissection (pelvic, para-abdominal aorta, presacral), resection of pelvic metastases, omentectomy, appendectomy, and hyperthermic perfusion chemotherapy for cancer. The postoperative pathological examination revealed the following findings: (1) (Bilateral) ovarian neoplastic lesions with microscopic tumor cells arranged in sieve-like, reticulum-like, solid sheets or tubules, with heterogeneous cells, prominent nuclei, and easily visible nuclear schizophrenic images, suggesting a diagnosis of Wolffian duct tumors. (2) Tumor infiltration was identified in intestinal surface nodules, pelvic masses, pelvic peritoneum, appendix, and greater omentum. (3) Lymph node metastases were detected below the renal artery and in the para-aortic region (2 out of 2 lymph nodes). Immunohistochemistry results revealed the following: CKpan (+), Vimentin (partially +), ER (++, about 30%), PR (++, about 15%), Calretinin (+), Inhibin α (a small amount of +), CD10 (+), β-Catenin (membrane +), and Ki-67 (+, 40%) as shown in **Figure 2**. The final diagnosis was a malignant tumor of the fallopian tube (stage IV).

## Diagnostic process

**Figure 3:**
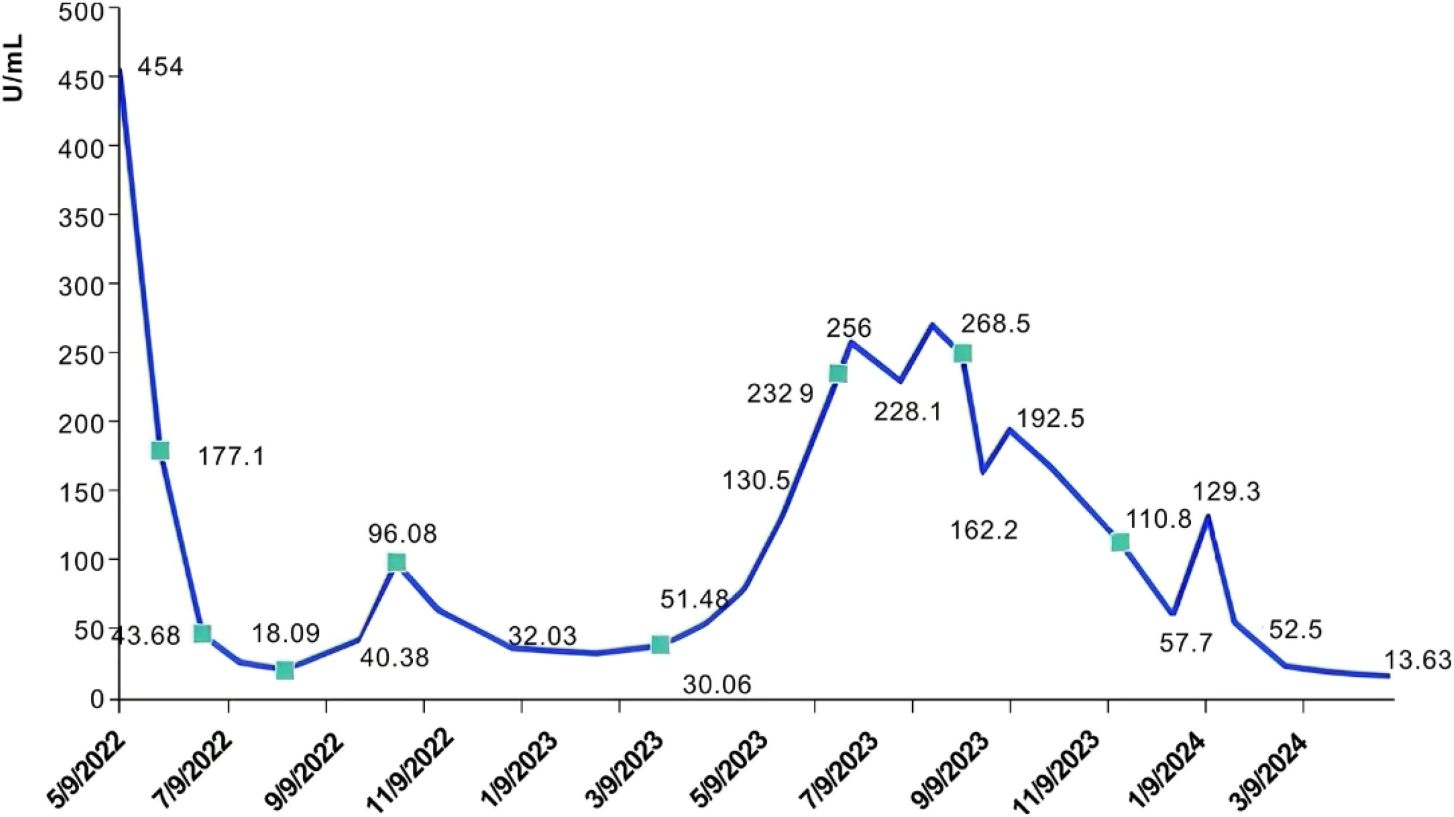
Curve of CA125 with treatment regimen. Tumor cell reduction (R1) was performed in May 2022, with the preoperative tumor marker value as the baseline. The treatment regimen included: (1) 1 course of treatment with “albuminpaclitaxel + carboplatin” followed by 2 courses of treatment with “albuminpaclitaxel + carboplatin + bevacizumab”; (2) 3 courses of “Domene + cyclophosphamide + bevacizumab”; (3) 5 courses of “Docetaxel + Nedaplatin + bevacizumab”; (4) 5 courses of “bevacizumab” maintenance treatment; (5) 3 courses of “gemcitabine + bevacizumab”; (6) 4 courses of Gemcitabine + oxaliplatin + Renvastinib; and (7) 4 months treatment with “Letrozole + lenvatinib”.

Following postoperative genetic testing, the results indicated 11 somatic cell variants, of which 9 were identified as clinically significant variants. These variants include CHEK1, NF2, ATM, STK11, ZNF217, BCL2L1, TP53, JAK1, and POLD1. The treatment options may involve chemotherapeutic drugs such as cyclophosphamide, fluorouracil, and platinum compounds. Targeted drugs such as temsirolimus (D), olaparib (C), antivascular agents such as bevacizumab, and endocrine therapies such as anastrozole and letrozole are considered effective. On June 1, 2022, the patient underwent 3 cycles of intravenous “albumin paclitaxel + carboplatin” and the second course of “bevacizumab” antiangiogenic therapy was initiated. After 3 cycles of chemotherapy, the tumor markers exhibited a continuous decline and the size of metastatic lesions improved (PR). However, due to intolerable side effects, the treatment plan was adjusted to include “dormethrin + cyclophosphamide + bevacizumab,” and after 3 cycles of chemotherapy, the levels of CA125, NSE, and SCC slowly increased. A whole-abdomen enhanced CT scan revealed new tumor metastases in the perihepatic peritoneum, the bilateral internal mammary arterial routes, and the angle of the heart and diaphragm. These findings indicate disease progression (PD), prompting evaluation of the treatment effectiveness. Due to disease progression and the patient’s positive status for ER and PR, the treatment regimen was changed in October 2022. The patient received 5 courses of intravenous chemotherapy consisting of “docetaxel + nedaplatin + bevacizumab” combined with antihormonal therapy using “anastrozole.” The review showed that the tumor size was similar to that of the previous one (SD) with no evident tumor size progression. However, due to severe side effects of chemotherapy that the patient could not tolerate, the treatment was transitioned to 5 courses of antivascular treatment with “bevacizumab” starting in March 2023. However, the tumor markers continued to rise, and new lesion progression (PD) was observed during the maintenance treatment phase as shown in **Figure 3**.

**Figure 4:**
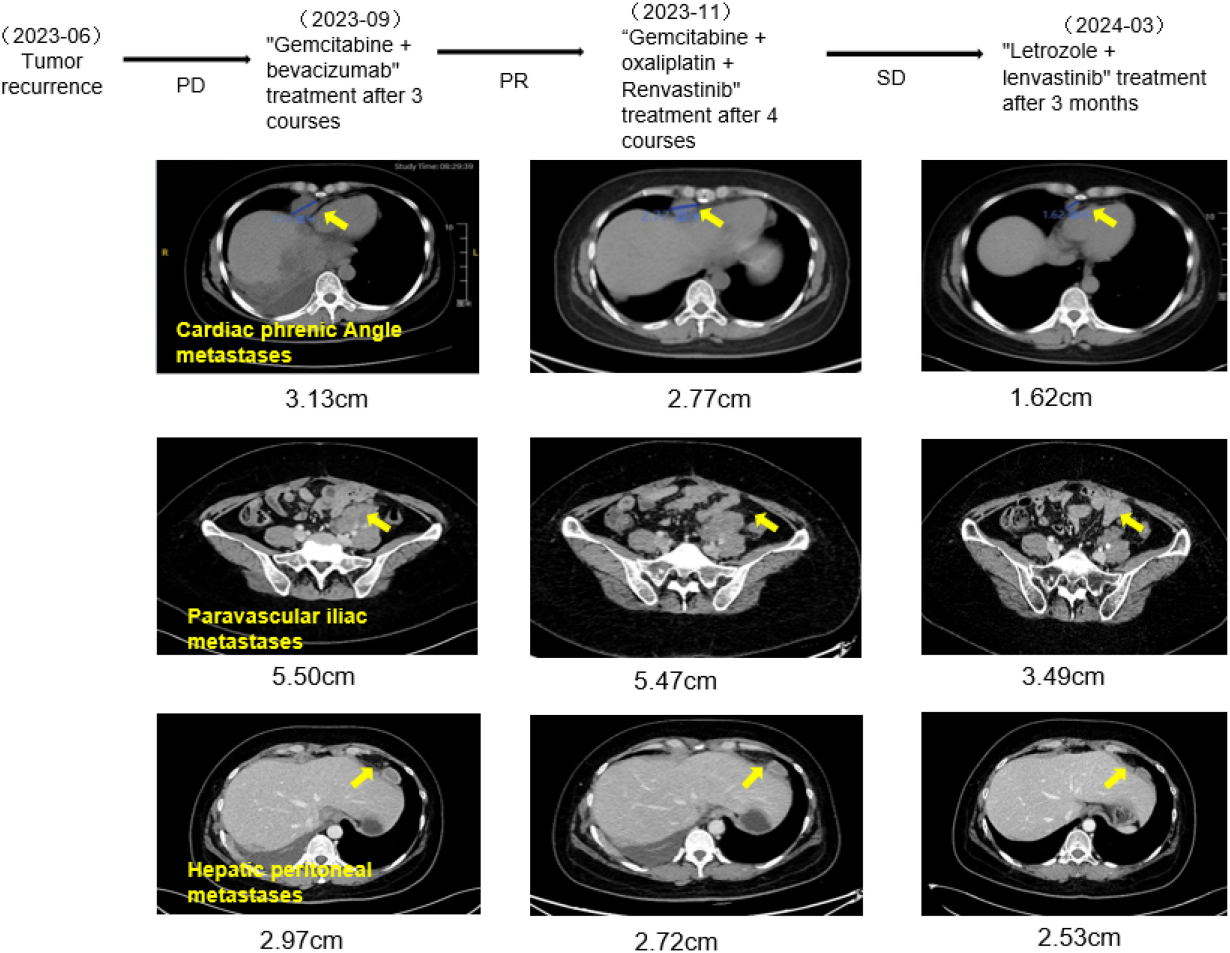
CT examination of the patient (the trend of lesion change with the change of treatment regimen).

In June 2023, the patient was admitted to the emergency room with chest pain, and a chest CT scan revealed pleural effusion, pleural metastasis, and peritoneal metastasis. Furthermore, the original metastatic foci in the pelvic and abdominal cavities had increased in size compared to previous assessments, indicating further tumor progression. After discontinuing anastrozole endocrine treatment, the patient underwent chemotherapy again in June 2023, given the use of platinum-containing drugs within the last 3 months, the patient was given 3 courses of treatment of gemcitabine on days 1 and 8 in combination with bevacizumab. However, subsequent evaluations indicated continued progression of the metastatic lesions (PD). On September 4, 2023, the treatment of “gemcitabine d1, d8 + oxaliplatin” was replaced, alongside oral lenvatinib at 8 mg once daily. After completing 4 cycles of this treatment, a CT scan indicated a partial reduction in metastatic lesions compared to previous assessments, and the tumor markers gradually declined, indicating a PR status to the treatment. However, the patient discontinued chemotherapy due to the side effects associated with the treatment. Considering the poor response to anastrozole, the patient’s treatment was adjusted on November 20, 2023, to oral maintenance therapy of lenvatinib 12 mg qd combined with letrozole 2.5 mg qd, and the patient has been treated with this regimen for 7 months, with the metastatic lesions progressively reduced, and the tumor markers, such as CA125, have continued to decrease to normal levels, with remarkable therapeutic effects as shown in **Figure 4**. The patient has now survived for 36 months since the onset of the disease and remains in continuous remission. He is advised to have regular reviews and follow-ups.

## Discussion

Wolffian tumor (WAT) is a rare gynecological tumor characterized by the simultaneous presence of various epithelial cells (1), occurring in the remnant tissue of the rich mesonephric ducts (2), mostly in the broad ligament, tubal mesentery, fallopian tubes, ovaries, and peritoneum (3), with malignant possibilities. This tumor generally expresses CK, calretinin, inhibin, CD10, and vimentin (4). However, the expression of ER and PR can vary from strong positive to negative. Due to the rarity of this tumor, there is no specific immunohistochemical staining to diagnose this tumor. Currently, the recommended treatment approach involves total hysterectomy along with double adnexal resection (5), which should be followed up closely for a long period in the clinic.

There are only one hundred cases of Wolffian tumors reported in the literature so far, and there is no uniform standard for clinical treatment. The patient in this case was young, and close observation was taken after the initial operation according to the pathology and the willingness to treat, but the tumor showed malignant biological behavior and recurrence. Based on genetic testing results and evaluation of clinical and test indicators, we found that the combination of platinum-based drugs with other chemotherapeutic drugs (6), such as albumin paclitaxel combined with carboplatin, docetaxel combined with oxaliplatin, etc., have shown favorable therapeutic effects. The antiangiogenic drug bevacizumab was integrated into the initial treatment regimen (a total of 18 courses of treatment). The therapeutic effects were greatly affected by adjustments made to the chemotherapy regimen during the combined application of these agents. The change in chemotherapy regimen affected the treatment outcomes in this case. Initially, the patient experienced disease progression shortly after switching to single-agent maintenance therapy with bevacizumab. Subsequent attempts to combine different chemotherapy agents did not yield satisfactory clinical results. Lenvatinib is a tyrosine kinase inhibitor with dual target inhibition properties (7–10), acting on vascular endothelial growth factor (VEGF), fibroblast growth factor (FGF), rearranged during transfection (RET), and other pathways. It can not only inhibit tumor angiogenesis but also regulate the tumor immune microenvironment to enhance the antitumor immune response (11). After the patient’s disease progressed, the combination of lenvatinib with chemotherapy led to significant remission, and the patient’s condition was ideally controlled. The patient is still in remission while receiving maintenance therapy with letrozole. It has been shown that endocrine therapy is indicated for patients with recurrent gynecologic tumors that express estrogen receptor (ER^+^) (12). In this case, the patient initially received a regimen combining anastrozole (13) with chemotherapy after experiencing progression on initial therapy. However, due to continued disease progression, this treatment was discontinued. Currently, the patient is receiving a maintenance regimen consisting of letrozole in combination with lenvatinib, which is clinically effective with no new signs of disease progression observed. This response was unexpected and we look forward to a more durable clinical benefit for the patient.

Therefore, for advanced or recurrent Wolffian tumors, a combination of platinum-based chemotherapy combined with antivascular drugs and endocrine therapy appears promising in providing patients with a longer survival benefit. However, further follow-up and collection of additional clinical data are essential to validate the long-term efficacy. Physicians should strive to achieve the best therapeutic outcomes by delaying tumor progression and extending patient survival.

## Data Availability

The raw data supporting the conclusions of this article will be made available by the authors, without undue reservation.
